# Detecting T-cell clonal expansions and quantifying clone survival using deep profiling of immune repertoires

**DOI:** 10.3389/fimmu.2024.1321603

**Published:** 2024-04-03

**Authors:** Anastasia V. Pavlova, Ivan V. Zvyagin, Mikhail Shugay

**Affiliations:** ^1^ Institute of Translational Medicine, Center for Precision Genome Editing and Genetic Technologies for Biomedicine, Pirogov Russian National Research Medical University, Moscow, Russia; ^2^ Shemyakin-Ovchinnikov Institute of Bioorganic Chemistry, Russian Academy of Sciences, Moscow, Russia; ^3^ Dmitriy Rogachev National Center of Pediatric Hematology, Oncology and Immunology, Moscow, Russia

**Keywords:** T cell repertoire analysis, adaptive immune receptor repertoire (AIRR), T cell receptor (TCR), clonal tracking, hematopoietic (stem) cell transplantation (HCST), immune reconstitution, TCR sequencing, immune monitoring

## Abstract

An individual’s T-cell repertoire constantly changes under the influence of external and internal factors. Cells that do not receive a stimulatory signal die, while those that encounter and recognize a pathogen or receive a co-stimulatory signal divide, resulting in clonal expansions. T-cell clones can be traced by monitoring the presence of their unique T-cell receptor (TCR) sequence, which is assembled *de novo* through a process known as V(D)J rearrangement. Tracking T cells can provide valuable insights into the survival of cells after hematopoietic stem cell transplantation (HSCT) or cancer treatment response and can indicate the induction of protective immunity by vaccination. In this study, we report a bioinformatic method for quantifying the T-cell repertoire dynamics from TCR sequencing data. We demonstrate its utility by measuring the T-cell repertoire stability in healthy donors, by quantifying the effect of donor lymphocyte infusion (DLI), and by tracking the fate of the different T-cell subsets in HSCT patients and the expansion of pathogen-specific clones in vaccinated individuals.

## 1 Introduction

The adaptive immune system is unique in its ability to recognize multiple previously unencountered pathogens and to form immunological memory of the encounter (1). T cells, which play a major role in antigen-specific immune response, represent a specific pool of clonally related cell lineages with unique antigen specificity (2). The repertoire of antigen-recognizing receptors of the T cells of an individual bears a unique fingerprint of past and ongoing immune challenges. This is because each T-cell clone is characterized by its own unique T-cell receptor (TCR) sequence that encodes its antigen specificity, tracking individual T-cell clones is a matter of studying the TCR repertoire (3). However, due to the large disparity in clonal size (i.e., the number of T cells comprising a T-cell clone), the huge diversity, and the high dimensionality of the TCR sequence space, such data have an inherently stochastic nature. The sequence diversity of TCR that enables its cognate antigen specificity is ensured by the mechanism of V(D)J rearrangement, which combines recombination events and random nucleotide insertions and deletions. This results in the unique nucleotide sequence of the complementarity-defining region 3 (CDR3) at the V(D)J junction that can be used to track individual T-cell clones. The theoretical number of sequence combinations is around 10^15^–10^20^ variants, but this becomes smaller (10^8^–10^9^) owing to the thymic and peripheral selection that deletes T cells with potentially autoreactive TCRs, while still providing enough variety to maintain an adequate immune response against virtually any encountered antigen (4). Therefore, determining the real changes in the T-cell repertoire structure from noise requires dedicated wet-lab protocols, highly accurate bioinformatic pipelines, and robust statistical methods (5).

A large number of recent works have employed immune repertoire profiling methods based on high-throughput sequencing techniques to study the dynamics of the T-cell repertoire by tracking the clonal emergence, survival, and expansion in response to various immune stimuli, such as vaccination (6) and hematopoietic stem cell transplantation (HSCT) (7), by monitoring minimal residual disease (8), detecting the autoimmune response (9), and assessing the aging of the adaptive immune system (2, 10). One of the most important applications of high-throughput TCR profiling in the coronavirus disease 2019 (COVID-19) pandemic era is the tracking of the response to pathogens such as severe acute respiratory syndrome coronavirus 2 (SARS-CoV-2), both in the course of infection, during follow-up, after infection, and during assay of the vaccination efficiency (11).

While multiple studies on T-cell clonotype tracking have been reported, there is no standard practice for the aforementioned issue, and the field still needs dedicated bioinformatic pipelines (12). Several statistical approaches for the assay of TCR frequency in the repertoire and of individual T-cell clonotype frequencies in donor blood samples have been described (12–15). Most of these approaches have been inspired by the problem of evaluating the species richness and diversity in the field of ecology, as covered by Colwell and Chao et al. (16), Efron and Thisted (17), and Hill (18). For example, in their attempt to infer factors underlying an unexpectedly broad distribution of naive T cells in human repertoire, de Greef et al. examined predictions based on neutral, power law, and log-linear models of species frequency distribution and compared them with experimental observations (19). Alternative strategies to account for sampling bias include utilizing spike-in TCR sequences in order to calibrate and assay the accuracy of a sequencing-based technique, thus estimating the probability of detecting a given TCR sequence in subsequent experiments (20). The aforementioned approaches are also linked to the concept of clonotype publicity and attempt to estimate the T-cell repertoire overlap between individuals and its reproducibility between replicates (21).

In the present paper, we propose a computationally simple but efficient approach that combines common repertoire and clonotype features, such as repertoire diversity and clonotype abundance, into a single statistical model that can be used to test hypotheses related to the T-cell clone expansion and survival of studied groups of T-cell clonotypes and entire samples. This model is highly accurate in recapturing the T-cell sampling process in both unperturbed and stimulated repertoires. We demonstrate this approach by detecting vaccination-driven clonal expansions, analyzing T-cell survival, and capturing differences in the T-cell subset behavior during HSCT.

## 2 Materials and methods

### 2.1 Model description

In the present study, we describe a model that statistically compares the clonotype sampling rates describing capture, survival, and expansion between conditions, clonotype subsets, and time points in a repertoire profiling time course. Firstly, the clonotype size group “**s**” was defined—singletons, supported by a single read/molecule [when using a unique molecular identifier (UMI)-based normalization]; doubletons, supported by two reads/molecules; tripletons, clonotypes with three reads/molecules and clonotypes that are highly expanded (i.e., more than three reads/molecules)—based on the clonotype frequency in the “pre” sample that can be either the preceding time point in a time course or the donor repertoire in clone tracking after HSCT, among others. The recapture probability in the “post” sample (the following time point in a time course, the recipient repertoire in HSCT, etc.) for a given group “**S**” of clonotypes was measured as *P* = *n*/*N*, where *P* is the capture probability, *N* is the number of unique clonotypes from group **S** in the “pre” sample, and *n* is the number of unique clonotypes from **S** found in both the “pre” and “post” samples. In the simplest case, **S** = **s**, but it can also incorporate other features, such as antigen specificity, **A**: **S** = **s** ⊗ **A**. Importantly, the proposed model includes the overall number of unique clonotypes in the “pre” and “post” samples, *N*_pre_ and *N*_post_, respectively. This model can also include various factors of interest, denoted as **G**, e.g., the HSCT protocol or the vaccination regime. Statistical analysis is subsequently performed using the linear model, log*P* ~ **S** + log*N*_pre_ + log*N*_post_ + **G**, as formulated in terms of the R programming language model definition to compare the effects of various factors.

### 2.2 Datasets used in the study

#### 2.2.1 HSCT dataset

The allogeneic hematopoietic stem cell transplantation (allo-HSCT) TCR repertoire data for donor lymphocyte infusion (DLI; *n* = 9) *vs*. non-DLI (*n* = 10) patients were obtained from Blagov et al. (7) and Zvyagin et al. (22). Briefly, the patients in this study received the αβT-cell depleted donor’s granulocyte colony-stimulating factor (G-CSF)-mobilized peripheral blood (PB) mononuclear cells (graft). After engraftment, a sub-cohort of patients (the DLI cohort) were infused with the donor’s CD45RA-depleted PBMCs at monthly intervals (a total of three times, with the last infusion on day 90 post-HSCT). Non-DLI patients did not receive the donor CD45RA-depleted PBMCs after HSCT (the non-DLI cohort). DLI cell samples from the DLI cohort or graft cell samples from the non-DLI cohort were collected to obtain the donor TCR repertoire. Recipient PBMCs for both cohorts were sampled at two time points: on day 120 and on day 360 post-HSCT.

To evaluate the clonal survival of T cells from distinct subsets, we also used previously unpublished data in the HSCT dataset from the study by Blagov et al. (7). These data represent the TCRβ repertoires of the CD4^+^ and CD8^+^ cell fractions obtained by fluorescence-activated cell sorting (FACS), as well as the central memory T (Tcm) and effector memory T (Tem) cells. These cell fractions were derived from recipient PB samples (CD4/CD8) or the DLI cell samples (Tcm/Tem and CD4/CD8). The cDNA TCRβ libraries for all samples in the HSCT dataset were prepared using the same method described in the next subsection.

#### 2.2.2 Vaccination dataset

Data on yellow fever virus (YFV) vaccination (“vaccination” dataset) were obtained from Pogorelyy et al. (6). Bulk PBMCs of three pairs of identical twins immunized with the YFV vaccine were collected at several time points: 7 days before vaccination; immediately after vaccination; and 7, 15, and 45 days after vaccination. The TCRβ repertoires were obtained for each sample as described below.

#### 2.2.3 Immune aging dataset

The repertoires for the “aging” dataset were taken from Britanova et al. (10). We focused on the T-cell repertoires of two healthy adult individuals. The PB samples were collected at two time points separated by 3 years. The two individuals were 27 and 47 years old at the first sample collection and, correspondingly, 30 and 50 years old at the second sampling.

### 2.3 TCRβ library preparation, sequencing, and repertoire data extraction

For all datasets used in the study, the TCRβ cDNA library preparation, sequencing, and data analysis were performed as previously described (23). Briefly, total RNA was extracted from cells using TRIzol reagent. The 5′ RACE cDNA libraries were prepared using primers specific for TCRβ constant regions. UMIs were introduced to cDNAs to allow removal of potential cross-sample contamination, to correct sequencing errors, and to normalize data (24). The cDNAs were processed using a two-step PCR amplification, and the libraries were then sequenced on the Illumina HiSeq 2500 platform.

Raw sequencing data were preprocessed and clustered by UMIs using the MIGEC software (24). Reads were mapped to the V, D, and J genes and the clonotypes assembled using the MiXCR software (25), generating datasets containing the read counts, the nucleotide and amino acid sequences of the CDR3 region, the V/J segment IDs, the sample ID, and related metadata for the identified TCRβ clonotypes. In this study, we defined TCRβ clonotype as a TCRβ sequence having a particular CDR3 nucleotide sequence in combination with an identical V segment ID.

The VDJdb database (26) was used to annotate the clonotypes specific for the YFV. In the “vaccination” dataset, we identified clonotypes that matched the TCRβ CDR3 associated with the A*02-LLW epitope in VDJdb as “YF-specific”. Based on previous findings on the sequence similarity of epitope-specific TCRs (27, 28), we allowed one mismatch (i.e., Hamming distance = 1) in the CDR3.

### 2.4 Statistical analysis and source code

All results were obtained using in-house R scripts and open-source R packages. All of the datasets used in this study are available at Zenodo (https://zenodo.org/record/7988170). The descriptive statistics of the samples used in the current study are presented in **Supplementary Table 1**.

R markdown notebooks reproducing the analysis described here can be found on GitHub (https://github.com/antigenomics/vdjtrack). The repository also contains an example dataset with the formatted T-cell repertoire sequencing data from the “vaccination” dataset, annotated with experimentally validated clonotypes that are induced by the vaccine (see “example/” folder), and an “example.Rmd” file that can be used as a template to run the VDJtrack pipeline in RStudio. The “code/” folder contains the code for preparing all the figures used in this work.

The framework was designed to work with next-generation sequencing data, including single-cell sequencing data from immune profiling. To perform analysis, the minimal input dataset should contain a list of clonotypes and their counts (number of UMIs/read counts). Clonotype can be defined as a unique CDR3 nucleotide or amino acid sequence. Alternatively, additional features, such as V, D, or J gene segments, can be added to the clonotype identifier. To check possible methods of converting data to the prerequisite format, one can refer to the “Load data” sections in the R markdown notebooks (see “example/” or “code/” folders on GitHub).

A file containing metadata, which includes the sample file names and their descriptions, can be used to simplify data preprocessing. Clone-specific or sample-specific features can be used to annotate different groups; for example, groups of clones can be annotated by antigen specificity, while groups of samples can be annotated by condition or by cell cluster in the case of single-cell data. The appropriate column should be added (see “vaccination dataset” as an example for grouping clonotypes by antigen specificity).

After organizing the data into the required structure, the next step is to track the clonotypes across time points and to group these clonotypes according to size (by read/UMI count). On the basis of clonal sequence identity, each clonotype in the combined dataset was labeled as either “found” or “not found” in the subsequent sampling (“post” time point) compared with the previous sampling (“pre” time point). All clonotypes were also categorized based on their size at the corresponding time point: as “singletons,” “doubletons,” “tripletons,” “large,” and “missing” for reads/UMI counts of 1, 2, 3, 4+, and 0, respectively.

Note that, subsequently, the beta distribution parameters can be estimated (posterior alpha and beta), and the capture probability *p* then calculated, which allows visualizing a probability distribution function using standard R graphical packages (see “DLI.Rmd” in the code section for an example). The beta distribution was fitted using the built-in “dbeta” function in R with Bayes (uniform) prior.

To quantify the effect of various factors (e.g., the number of clones detected in the “pre” repertoire, the number of clones detected in the “post” repertoire, the clonotype group size, and other grouping factors) on the recapture probability, a log-linear model was used to determine the log-transformed coefficients and their *p*-values. To assess the linear models and quantify the significance of the various model parameters, ANOVA was performed using the corresponding built-in R functions.

### 2.5 Inferring expanded clones using “edgeR”

A classic “edgeR” approach (as described in the edgeR User’s Guide) was used (29) for the comparison of the bulk TCRβ repertoires of six individuals on day 15 and on day 45 after vaccination. Reads with mean counts across pairwise comparisons of <4 were filtered out. The TTM (trimmed mean of *M* values) method was used for data normalization, and the dispersion was estimated using the quantile-adjusted conditional maximum likelihood (qCML) method. To determine the significantly expanded clonotypes between two time points, an exact test for the negative binomial distribution to compute exact *p*-values was used. Clonotypes with a log2 fold change ≥5 between two time points and a *p*-value ≤0.01 were considered as significantly changed and, thus, vaccine-associated.

## 3 Results

### 3.1 Model description and rationale

**Figure 1:**
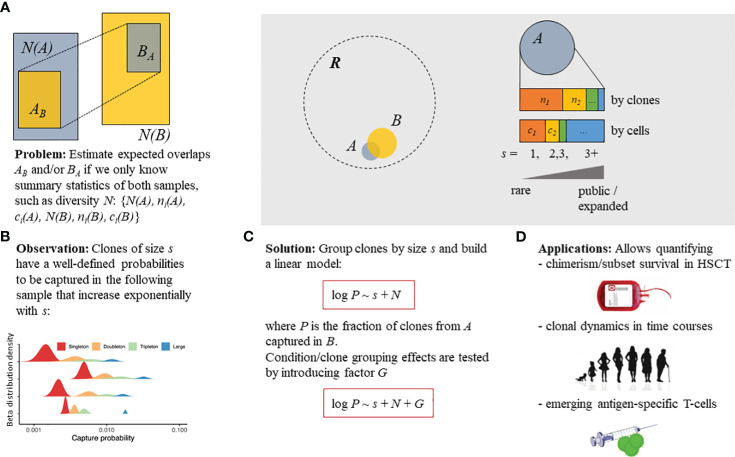
Overview of the Т-cell clone tracking model. **(A)** Statistical model described in the present study that aimed to estimate the probability of capturing a T-cell receptor (TCR) clonotype belonging to a subset of interest (sampled at the “pre” time point, repertoire A) in a subsequent sample (“post” time point, repertoire B). **(B)** Three parameters describing the TCR clonotype sampling process: clonotype size (*s*) [singleton—supported by a single read or unique cDNA molecule [identified on the basis of a UMI; doubleton—supported by two reads/cDNAs, etc.]; the number of clonotypes in the original sample, *N*(*A*); and the number of clonotypes in the subsequent sample, *N*(*B*). **(C)** Proposed simple log-linear model that can incorporate and estimate the effect of clonotype/sample grouping factor *G* on the capture probability. **(D)** The model can be applied to detect differences in cell survival post-hematopoietic stem cell transplantation (HSCT), the normal clonal dynamics in aging, and the emerging clonotypes induced by vaccination. Linear models (*red boxes*) are given in terms of the R programming language formula specification. *R* denotes the overall existing T-cell repertoire diversity, while samples (i.e., A or B) represent subsets [with *N*(*A*) or *N*(*B*) total clonotype number, respectively] that can be partially overlapped.

In the present study, we aimed to quantify the expected number of T-cell clones or clonotypes present in the original sample (“pre” time point) that were successfully captured in a subsequent sample (“post” time point), with the ability to compare the capture rates between groups of clones or clonotypes and experimental conditions (**Figure 1**). In repertoire sequencing, the T-cell clones can be defined by identical nucleotide sequences of their TCR genes (both TCR α and β chains). However, as most of the repertoire sequencing data available to date, as well as the data used in our study, represent the sequencing data of only the TCR β chains, we further used the term “clonotype” to designate the TCRs that came from a group of T cells with identical TCR β chains. Both “clone” and “clonotype” will be further used synonymously to refer to clonotype as the model used can be applied for both paired (α and β) and unpaired (α or β) TCR repertoire data. We have empirically determined that the capture rate is dependent on the size of the clonotype and the total number of clonotypes in the original and subsequent samples, as shown below.

**Figure 2:**
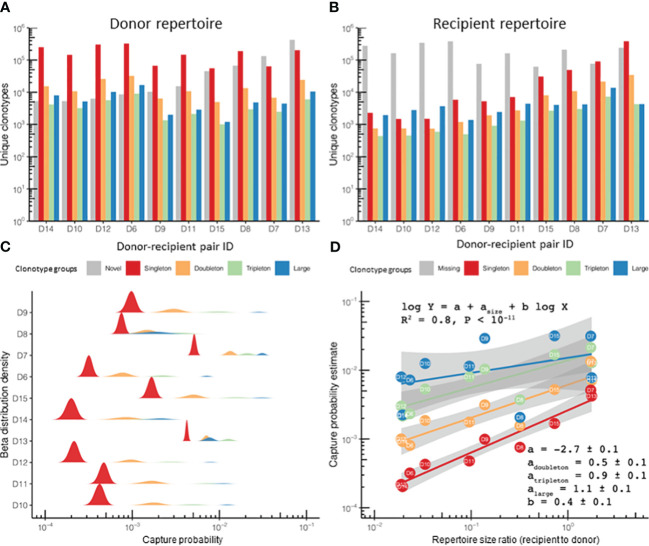
The clonotype recapture probability during immune reconstitution after hematopoietic stem cell transplantation (HSCT) was defined by the clonotype size in the original sample at the “pre” time point and the repertoire diversity. **(A, B)** Number of unique clonotypes in the donor and recipient repertoires (*n* = 10) assigned to size groups based on the number of corresponding TCRβ cDNAs identified for each clonotype: *single* (singletons), *two* (doubletons), *three* (tripletons), and *4+* (large) in repertoire sequencing data [“HSCT” dataset; data from donor–recipient pairs transplanted in the non-donor lymphocyte infusion (non-DLI) setting are shown]. The plot also shows the number of clonotypes in the recipient, but not in the donor [“novel,” *gray bars* in **(A)**], or found in the donor, but not detected in the recipient repertoire [“missing”, *gray bars* in **(B)**]. Note that the “novel” clonotypes in panel **(A)** and the “missing” clonotypes in panel **(B)** are depicted for comparison and represent the clonotypes detected only in the recipient (“novel”) or the donor (“missing”) repertoire, respectively. **(C)** Recapture probability for a clonotype belonging to each of the four size groups in the donor. The probability distribution is modeled using beta distribution with the number of detected clonotypes and the number of missing clonotypes as parameters. A ridgeline plot is used to visualize the distributions: each sample has an equal share of space in the general *Y*-axis, and individual *Y*-axes are scaled to fit the highest peak of corresponding density distributions. **(D)** Log-linear model fitting clonotype recapture probability (*Y*-axis) based on the clonotype size in the donor sample (*points* and *lines of different colors*) and the diversity of donor and recipient samples (ratio plotted on the *X*-axis). Adjusted *R* ^2^ for the entire model and corresponding linear fit parameters and SDs are provided in the main text. Clonotype size colors in **(C, D)** are the same as those in **(A)**.

Our model stems from observations of T-cell clonotype tracking in the HSCT data (**Figure 2**), where the donor repertoire is considered as the original sample (“pre” time point) and the corresponding recipient post-HSCT repertoire is considered as the subsequent sample (“post” time point). This allows monitoring the survival of the donor T lymphocytes probed by the donor repertoire. Clonotype abundance was binned into size groups according to the number of TCRβ cDNAs identified for a given clonotype: singletons, doubletons, tripletons, and large (expanded) clonotypes (**Figures 2A, B**). This abundance-based approach was previously introduced for the estimation of species diversity and number of unseen species by Colwell et al. (16) and was implemented in a number of immune repertoire sequencing data analysis software packages to quantify immune repertoire diversity (14). Note that, due to differences in the number of cells, messenger RNA (mRNA) molecules, and sequencing depth, the sample sizes can vary a lot, which makes their direct comparison impossible without proper normalization, but which was handled by our model as described below.

In our examples for all clonotype size groups, we observed around 1,000 clonotypes and more. It is noticeable that the group of missing clonotypes (detected in the donor but not seen in the recipient) was substantial (**Figure 2A**): in the case of sampling of the recipient PBMCs on day 60 after αβT/CD19-depleted HSCT, a high rate of detectable clonotypes was not expected in the recipient due to sampling effect and the long period of T-cell repertoire reconstitution (22). While quantitatively most of the clonotypes surviving post-HSCT were singletons (**Supplementary Figure 1A**), the proportion of recaptured clonotypes originating from the donor was remarkably higher in the large or tripleton size groups (**Supplementary Figure 1B**). Thus, expanded clonotypes have a better chance of surviving the procedure compared with those supported by a few TCRβ cDNAs, i.e., T cells.

We computed the capture rate of a clonotype group, i.e., clonotypes having the same size in the “pre” repertoire (i.e., in the donor sample), as the fraction of clonotypes from that group that were present in both the “pre” and “post” (i.e., in the recipient) sample time points. When fitted with beta distribution, the capture rates of the clonotypes were nicely ordered by abundance in log scale (**Figure 2C**), while the remaining difference in the sampling can be explained by the difference in the donor and recipient repertoire diversities, leading to a robust log-linear fit (**Figure 2D**). Beta distribution was chosen as a natural method (from the Bayesian point of view) for visualizing uncertainty in the clonotype capture rate by learning from data, but which was not directly used in the modeling, which used log transformation for frequencies. The model can then be supplemented with a grouping variable in order to measure the significance of its input: the donor-specific features [e.g., the donor diagnosis, the cytomegalovirus (CMV)-positive/negative status, the HSCT protocol with or without additional infusions of donor T cells, the organ/tissue type, or the cell subpopulation as the repertoire source, among others] and the clonotype-specific features, such as antigen specificity, can be compared across different time points.

### 3.2 Longitudinal repertoire stability and sampling model

**Figure 3:**
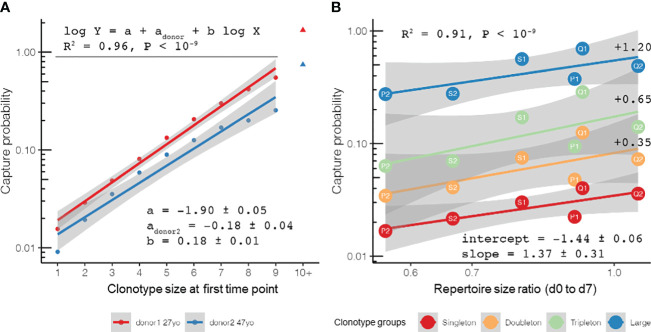
Repertoire stability and clonotype sampling model in time course setting: the probability to sample a clonotype at a later time point given its observed size at a previous time point and repertoire diversity at both time points. **(A)** Log-linear fit of probability (*Y*-axis) to detect clonotypes supported by different numbers of TCRβ cDNAs [unique molecular identifiers (UMIs), *X*-axis] in samples taken 3 years apart in two healthy donors of different ages (27 and 47 years old at the first sampling) [data from the “aging” dataset of Britanova et al. (10)]. The difference in the intercept of these curves can be attributed to the different diversity ratios of the post-to-pre samples of two donors [0.42 for 30 years old (“post” time point) *versus* 27 years old (“pre” time point) and 0.14 for 50 years old (“post” time point) *versus* 47 years old (“pre” time point)]. **(B)** Probability to capture clonotypes sampled a week before vaccination (day −7, “pre” time point) and on the day of vaccination (day 0, “post” time point) in *n* = 6 sample pairs [data from the “vaccination” dataset of Pogorelyy et al. (6)] fitted with a log-linear sampling model. **(B)** The values of the size-dependent coefficients are shown to the *right of the corresponding lines*.

We proceeded with our baseline repertoire sampling model by analyzing the repertoire changes with time in healthy adult donors using data from the “aging” study of Britanova et al. It has already been shown that human T-cell repertoires are extremely stable, even when sampled over a 3-year period (10). In this study, we showed that the suggestion to split clonotypes into groups based on their size (which lies in the basis of our model as described in *Materials and methods*) can recapture T-cell clonotype sampling behavior on large timescales, and our observations revealed that most of the differences between the TCR repertoires obtained from the same donor across a 3-year period can be explained by random sampling. Owing to the depth of repertoire sequencing in the “aging” dataset, one can observe a clear log-linear dependence between the clonotype size and sampling probability up to clonotypes supported by 10+ reads when the stochasticity of large clonal expansions is in effect (**Figure 3A**). This observation can be used to justify the rarefaction approaches common in the field of ecology for estimating the species richness of the TCR repertoires of PBMCs or T-cell subsets (14, 16). The population (repertoire) frequency of a clonotype from a group of a given size can be estimated, assuming that the capture probability can be derived from Poisson distribution as *P*_capture_ = 1 − exp(*−ϕ* × *R*), where *R* ~ 10^6^ is the total number of T cells (TCRβ cDNA molecules, UMIs) in the sample, arriving to *ϕ* ~ 10^−8^ (see the example R script referenced in *Materials and methods* for calculation). This estimate was weighted to account for the different fractions of clonotypes represented by different numbers of cells: fractions of singletons, doubletons, and tripletons, among others, based on the count of UMIs identified for each TCR clonotype in the repertoire. Given ~10^11^ T cells in adult PBMCs (30), the size of a T-cell clonotype can be roughly estimated as ~10^3^ cells, in agreement with earlier observations that gave the lower bound of the number of unique T-cell clones as ~2.5 × 10^7^ (31). Interestingly, in-depth analysis using rarefaction curves (14) showed that, while the repertoire becomes less diverse with age, the sets of clonotypes captured at both time points in an individual tend to become richer with age in terms of capture probability, a likely manifestation of the increase of the memory T-cell compartment (**Supplementary Figure 2**).

Moreover, we examined the repertoire stability on the timescale of a single week using the YFV vaccination dataset from the study by Pogorelyy et al. (6). We compared the individual repertoires sampled in a week before the vaccination (day −7, the “pre” time point) and those sampled just on the day of vaccination (day 0, the “post” time point). As can be seen in **Figure 3B**, the log-linear model accurately predicted sampling in all six donors in the study: the clonotypes for which two unique TCRβ cDNAs were identified (doubletons) were ~2.2 times more likely to be captured than the clonotypes represented by a lower number of T cells (singletons), while tripletons were ~2.0 times more likely to be captured than doubletons. The expanded clonotypes were mostly persistent (with a capture probability close to around 10%–50%) and were ~3.5 times more frequently captured than tripletons. Again, the two factors that explained the most variance in the clonotype recapture probability were the size of the clonotype (*p* < 0.001, 10^−6^, and 10^−11^ for the doubleton, tripleton, and large clonotypes, respectively) and the ratio of repertoire diversity (*p* < 0.001) in two subsequent sampling points, yielding a log-linear fit with an adjusted *R* ^2^ = 0.91.

### 3.3 Vaccination time course and emergence of antigen-specific TCRs

**Figure 4:**
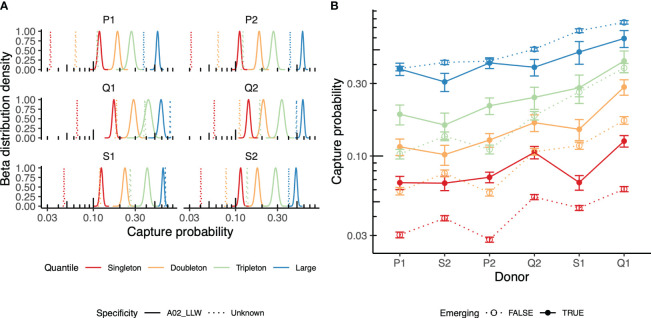
Repertoire behavior in the vaccination time course. **(A)** Recapture of the yellow fever virus (YFV)-specific T-cell receptors (TCRs, *bold*) in the post-vaccination peripheral blood (PB) repertoires of six individuals. Beta approximation for the distribution of the capture probability on day 45 (“post” time point) of the TCRs identified on day 15 (“pre” time point) is shown. The TCRs annotated as HLA*02-LLW-specific TCRs (*solid*) are compared to the rest of the TCR clonotypes of the repertoire on day 15 (*dashed line*). Clonotypes were grouped based on their size in the “pre” time point: singletons, doubletons, tripletons, and TCRs supported by four or more UMIs (large). **(B)** Evaluation of the recapture of “emerging” TCRs, i.e., those not present on day 0 (before vaccination), but present on day 7 after vaccination in the donor repertoire. The recapture probabilities between the emerging (*solid points* and *lines*) and persistent (*dashed lines*) TCRs falling into different groups of clonotypes (singletons, doubletons, tripletons, or large) are compared for pairs of repertoires sampled on day 15 [vaccination response peak (“pre” time point) *vs*. day 45 (post-vaccination, “post” time point)].

Prior knowledge of TCRs specific to antigens of interest obtained using techniques such as tetramer-based sorting followed by repertoire sequencing (32) or annotating the list of TCRs using a database of TCRs with known specificity, such as VDJdb (26), allows identifying the specific TCRs that were induced in the course of vaccination. Here, we applied prior knowledge of TCRs cognate to the YFV A*02-LLW epitope to trace specific clonotypes in the course of vaccination [data from Pogorelyy et al. (6)]. TCR sequencing of various antigen-specific T cells isolated using the peptide-bound major histocompatibility complex (pMHC) multimer technology showed high CDR3 sequence similarity among the clonotypes specific to the particular pMHC (27, 28). Based on this, we annotated all clonotypes in the dataset that matched to or differed by a single mismatch in their CDR3 region from the known A*02-LLW-specific TCR sequence as “specific.” Our approach indicates that “specific” clonotypes observed on day 15 (peak vaccination response, “pre” time point) were persistent in the repertoire on day 45 (“post” time point) and were more likely to be captured than other clonotypes (**Figure 4A**). This can be explained by the persistence of vaccination-induced memory T cells specific to the antigen.

Moreover, TCRs emerging on day 7 post-vaccination, but not present prior to vaccination, were more likely to be persistent across days 15 and 45, suggesting their involvement in vaccine response (**Figure 4B**), further supporting the ability of the proposed approach to detect vaccine response. Note that these observations were only partially true for the expanded clonotypes that were already present in the donor. These can be attributed to the bystander activation of memory T cells (33) and/or to the presence of a cross-reactive response to similar antigens.

We reproduced the main results on the recapture probability for the expanded/vaccine-induced clonotypes using an edgeR-based approach (see *Materials and methods*). “edgeR,” an R package developed for differential expression analyses of transcriptome data, was used for the inference of clonotype expansion previously (6). The expanded clonotypes identified by the edgeR-based approach had a higher recapture probability (**Supplementary Figure 3**, solid lines are shifted to the right along the *X*-axis for all group sizes), showing the good agreement of our model with the orthogonal method.

### 3.4 HSCT conditioning and donor T-cell recovery

**Figure 5:**
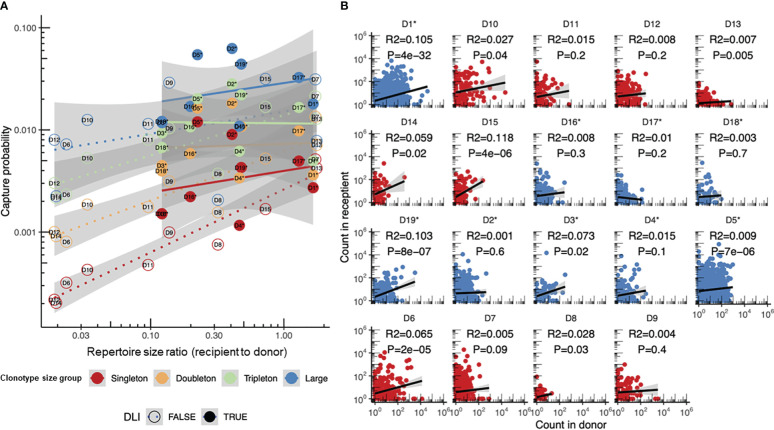
Donor clonotype survival in recipients transplanted with and without additional infusion of donor memory T cells (donor lymphocyte infusion, DLI). **(A)** Comparison of the sampling probabilities for clonotypes of different sizes using the log-linear model proposed in this paper. DLI samples (*n* = 10, the same as in **Figure 2D**) are shown with *dashed lines* and *circles*, while non-DLI samples (*n* = 9) are shown with *dots* and *solid lines*. Non-DLI samples have significantly higher log recapture rates when corrected for clonotype size and sample diversity, with estimated increases in the log capture probability of *p* = 0.30 ± 0.07, *p* = 3 × 10^−4^. **(B)** Comparison of the clonotype frequencies in the donor [GM-CSF-mobilized PB (non-DLI) or CD45RA-depleted PB T cells (DLI)] and recipient repertoires post-hematopoietic stem cell transplantation (HSCT). The donor clonotypes in the DLI and non-DLI samples are shown in *blue* and *red*, respectively. *R* ^2^ estimates and *p*-values are provided for each plot. *Asterisks* denote the DLI samples.

We further utilized our statistical approach to study the differences in the donor T-cell clonotypes post-HSCT survival depending on the HSCT setting. As before, the donor repertoire was denoted as the “pre” time point and the recipient repertoire sampled after HSCT as the “post” time point. The analysis of the different types of transplants used for HSCT revealed substantial differences in the DLI and non-DLI samples (**Figure 5A**). While the capture probability of the T-cell clonotypes from transplants containing the unperturbed TCR repertoire (non-DLI samples) resulted in overall agreement with the log-linear model as shown previously, the transplants depleted for CD45RA^+^ cells (DLI samples), i.e., mostly consisting of memory T cells, showed little dependence on the donor and transplant repertoire diversity, but overall had a higher survival rate, as expected from previous observations (7). Notably, while conventional analysis of the correspondence between the clonotype frequencies in the donor and transplant showed some significant correlations (**Figure 5B**), the overall correlation coefficient values (effect sizes) were low, and there were no differences between the DLI and non-DLI samples.

**Figure 6:**
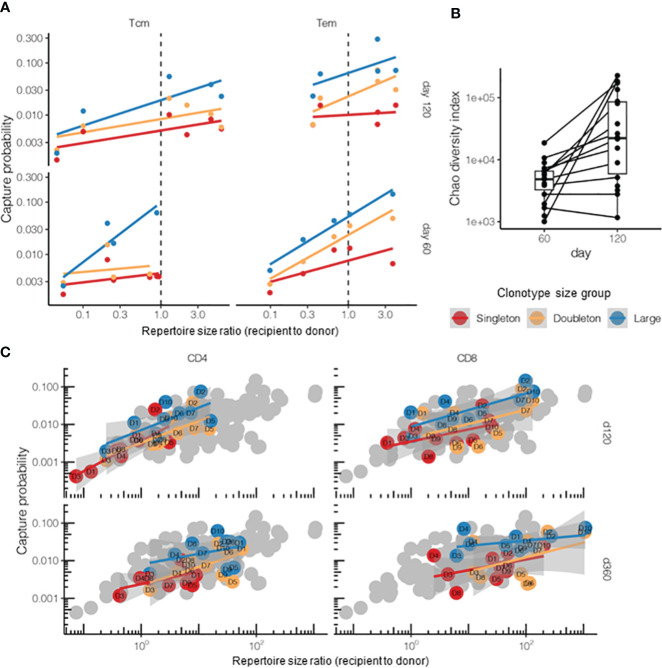
Post-hematopoietic stem cell transplantation (HSCT) survival of the donor T-cell clonotypes from different T-cell subpopulations. **(A)** Survival of the donor central memory T- (*Tcm*) or effector memory T-cell (*Tem*) clonotypes on days 60 and 120 after HSCT (*n* = 5 donor–recipient pairs, “post” time points). The donor lymphocyte infusion (DLI) Tem or Tcm repertoires were used as the “pre” time points. The overall log-linear model had an adjusted *R* ^2^ = 0.38 and *p* = 10^−6^. Tem survived better in terms of the number of donor clonotypes compared with Tcm (*t* = 3.01, *p* = 0.004, *post-hoc t*-test for log values). **(B)** Days 60 and 120 post-HSCT recipient repertoire diversity normalized to analysis depth and measured using the Chao diversity index (*n* = 25 patients). The log diversity index was significantly higher on day 120 compared with that on day 60 using a two-sided *t*-test (*t* = −3.95, *p* = 0.0006). Note that most recipients were measured at only one time point. **(C)** Comparison of the survival of CD4^+^ and CD8^+^ donor Т cells on days 120 and 360 post-HSCT. The donor DLI CD4^+^ or CD8^+^ repertoires served as “pre” and the corresponding recipient CD4^+^ or CD8^+^ repertoires (sorted either on day 120 or day 360) as “post” time points. *Gray dots* represent all samples from the HSCT dataset for comparison. The overall log-linear model had an adjusted *R* ^2^ = 0.56, *p* < 10^−16^. No significant difference between the cell subsets was observed (*p* = 0.89). Note that this plot had no tripletons and merged into the “large” quantile due to the low clonotype counts of the sorted T-cell populations.

The survival rates for the T cells from the two main memory subsets of donor cells, i.e., Tcm and Tem T cells, showed differences in the DLI setting of HSCT (**Figure 6A**). As the numbers of sorted T cells were relatively low, we focused on singletons and doubletons, combining tripletons with the “large” clonotypes. Overall, there was good agreement with the log-linear sampling model, and Tem cells showed higher survival probability on day 120 post-HSCT (*t* = 3.01, *p* = 0.004, *post-hoc t*-test for log values). It should be noted that the increased capture rates in both subsets on day 120 can be attributed to the continuing clonal proliferation and reconstitution of the overall T-cell count and the repertoire diversity (**Figure 6B**) reported previously for this period after TCRαβ/CD19-depleted allo-HSCT for pediatric patients (22, 34).

Using the same logic, we compared the CD4^+^ (helper T cells) and CD8^+^ (cytotoxic T cells) donor T-cell survival on days 120 and 360 post-HSCT, but did not observe any significant difference between them, while the model based on clonotype size and sample diversity ratios showed a fine correlation (**Figure 6C**). Note that, for the cell subsets, we limited the analysis to singletons and doubletons and merged the tripletons into the “large” quantile as the number of sorted clonotypes was too small for proper quantification.

## 4 Discussion

The immune repertoire of an individual is extremely diverse, and existing methods [i.e., adaptive immune receptor repertoire sequencing (AIRR-seq) and single-cell RNA sequencing (scRNA-seq)] reveal only a minor fraction of the immune receptors present in an individual. This results in sparse datasets, causing complications in T-cell clone tracking and in comparative analysis of the immune repertoires (35). In general, it is almost impossible to determine whether a certain rare TCR is missing in the dataset by chance or is lost due to the extremely low number of T cells supporting this clone in an individual (8, 36).

In the present work, it was demonstrated that, even with the aforementioned challenges, it is still possible to quantify the incidence of certain clones in donors using a relatively straightforward statistical approach. It is challenging to construct a proper statistical model for a single T-cell clonotype: factors such as high stochasticity due to the rarity of clonotypes and the “noise” from the sequencing library preparation steps play a role. The main idea behind our approach is that an accurate model can still be built when considering a set of hundreds or more T-cell clonotypes associated with a homologous TCR sequence or from sample-specific categories, such as the cell subset or the origin group (e.g., donor-derived in HSCT). We measured the recapture probability in the “post” sample for a given group of clonotypes identified in the “pre” sample using the repertoire diversity and clone size. Beta distribution was used to visualize uncertainty in the clonotype capture rate, but was not used directly in the modeling, which used log transformation for clonotype frequencies. The log-linear model was used as it is one of the simplest yet most efficient ways to explain variance in the data and to not overfit the data: basic model parameters are limited to factors such as clonotype size and repertoire size. This model can be easily extended with arbitrary grouping variables specified by the user, and it is robust enough to handle different types of datasets, including ones with low coverage and with a low number of cells. More sophisticated models that handle the underlying clone size distribution can be implemented but would require deep repertoire sequencing with a control for the number of events (mRNA/reads) per cell.

Alternative methods have already been described for T-cell clone size estimation [for example, see de Greef et al. (19)]. However, other approaches are focused on different aspects. De Greef et al. modeled the frequency of naive T cells from generation (VDJ rearrangement) probabilities (*P*_gen_) and highlighted several cases of frequent rearrangements that fall out of the model. Moreover, all the work was performed by separating the CD4 and CD8 memory and naive subsets, which behave differently. The comparisons were done between the real repertoire frequency distribution and the model built from *P*_gen_ that can be adjusted to account for thymic selection (but not for memory expansions). In this paper, we proposed a method to evaluate the differences in the clonotype survival rates. These are dependent on the real clonotype frequency, which is extremely hard to measure as most clonotypes are rare and 1 cell per 10,000–1,000,000 can represent either naive or memory cells. Moreover, *P*_gen_-based models can, strictly speaking, only be used for T-cell repertoires before thymic selection. In our case, we are agnostic to the *P*_gen_ and cell subset (which can be used as a comparison factor) and group clonotypes by their observed counts, expecting that, in general, they will behave similarly during sampling and that any biases can be attributed to changes in survival, expansion, and other probabilities.

As the top of the immune receptor repertoire is, in general, highly stable (10, 37–39), and on that scale most of the changes associated with rare clone incidence are due to sampling effects, we were able to show that our model can accurately predict the probability to recapture a T-cell clonotype of a certain size (supported by a certain number of reads/UMIs, i.e., supported by a certain number of T cells) over the course of several weeks and even years. The model parameters can be easily determined and fine-tuned for a specific AIRR-seq protocol.

In the section on YFV, we noted that antigen-specific clones are more likely to be captured on day 45 post-vaccination; however, as shown in **Figure 4A**, the capture probability difference between the specific and nonspecific large clonotype groups was very small, particularly for Q1, Q2, S1, and S2. As the recapture probability is directly dependent on clonal size, large clonal expansions have high recapture rates disregarding their relatedness to YFV even in the context of vaccination. Large expansions in the CD8 subset are well known for T-cell clones specific to persisting viruses, such as CMV or Epstein–Barr virus (EBV). The results of this paper showed the stability of large clones during the 1-year timeline; similarly, in a number of previous studies, the stability of large clonal expansions has been demonstrated (10). Even after an acute antiviral response or vaccination, only a few T-cell clones can reach comparable cell numbers when using bulk TCR repertoire sequencing and ordinary (5–20 min) sample size. During the immune response, most “events” in terms of changes in the clonal abundance are usually observed for small- or medium-sized clones in PB samples (11). Indeed, our model showed more differences for the smaller groups compared with the large groups. There was a relatively small number of YF-specific clones falling into the group designated as “large” in this study, and the most non-YF-specific T-cell clones from the group were also recaptured with high probability, leading to small differences in the recapture probability between the YF-specific and the rest of the large clones. Depending on the aims of a particular study, the repertoire can be more precisely dissected, allowing a detailed study of this part of the repertoire.

We further applied our approach to study the survival of donor T cells in recipients after allo-HSCT. Even in cases where the majority of donor T-cell clonotypes could not be detected in the PB of patients after TCRαβ/CD19-depleted HSCT (22), the recapture probability of the traceable clonotypes accurately follows our model. This allowed us to compare alternative approaches to allo-HSCT with the clonotype recapture probability inferred by our model and to detect differences in the survival of donor T cells from different cell subsets. Our results supported the initial observation that the clonotypes from memory-enriched donor T-cell infusions survive in recipients’ blood (7) and demonstrated differences in the survival rates of the clonotypes associated with the central memory or effector memory phenotype, as well as the absence of such differences between CD4^+^ and CD8^+^ donor T cells. It has to be noted that, due to the inability to sequence all of a recipient’s T cells, the better recapture rate in the subsequent sample of a given size could also reflect a higher expansion rate rather than the complete elimination/persistence of a clonotype on a periphery.

Comparison of the serial time point repertoires in the vaccination dataset allowed inferring the groups of clonotypes with homologous TCRs induced by YFV vaccination that are likely to be specific to viral antigens. This result was validated using the TCRs that were previously shown to be specific to the YFV antigen by tetramer staining. Overall, the results of the repertoire analysis using our statistical approach complement the conclusions of the study in which the dataset was obtained (6).

Having ground truth values for clonotype frequencies is close to impossible due to the extreme diversity of the T-cell repertoire and the limited sampling [a common challenge in analysis that involves undetectable species; see (37)]. To further validate our approach, we used the YFV vaccination data and edgeR as a method with different underlying statistical basis to identify the clonotypes with increased cell counts between two time points (6). In this dataset, the vaccine-induced clones were independently verified and were known to expand and have a characteristic trend in their frequencies. This trend in frequencies is in a good agreement with that of our model (**Supplementary Figure 3**).

Our approach can be easily generalized to B-cell AIRR-seq data. Interestingly, as B-cell receptors naturally cluster into tree-like structures (i.e., “clonal groups”) as a result of somatic hypermutation, the definition of a functional group in this case is relatively simple. Using our approach, it is possible, for example, to monitor a single large tree/clonal group formed by an antigen-responding B-cell clone in dynamics and observe how its expansion changes in a time course (40).

Another natural application is the analysis of single-cell transcriptome data completed with VDJ sequencing. In this case, functional groups of T cells can be introduced by clustering the gene expression profiles of single cells and defining the T-cell phenotypes. By tracking the TCRs corresponding to a certain phenotype, our method can be applied to identify the T-cell populations that undergo expansion and depletion across various conditions and tissues. Given the wealth of single-cell sequencing data that incorporate TCR sequencing, this approach appears to hold a lot of promise and can be used to reanalyze existing data.

Other potential applications of our approach include basic immunology studies such as quantifying clonal selection and the diversity of adaptive immune repertoire in various setups (2, 19, 41), monitoring rare groups of clonotypes involved in autoimmune responses (9), and tracking the fate of T cells in the course of cancer immunotherapy by comparing the TCR repertoires of various cell subsets and post-therapy time points (42, 43). Our approach can be easily incorporated into routine data analysis pipelines, both for AIRR-seq data and other types of datasets, such as scRNA-seq. Overall, we believe that our approach can be useful for both basic and applied research and that it has certain potential for clinical applications.

## Data availability statement

All datasets used in this study are available at Zenodo (https://zenodo.org/record/7988170). R markdown notebooks reproducing the analysis described here can be found on GitHub (https://github.com/antigenomics/vdjtrack).

## Ethics statement

Ethical approval was not required for the studies involving humans because in this study we used previously published datasets or additional datasets from these original researches. Ethical approval statements for collecting samples to generate datasets used in the present study are provided in the original research papers. The studies were conducted in accordance with the local legislation and institutional requirements. Written informed consent for participation was not required from the participants or the participants’ legal guardians/next of kin in accordance with the national legislation and institutional requirements because in this study we used previously published datasets or additional datasets from these original researches. The study did not involve any other participants except of authors of the manuscript clearly provided their agreement by submitting it.

## Author contributions

AP: Investigation, Software, Visualization, Writing – original draft, Writing – review & editing. IZ: Conceptualization, Data curation, Funding acquisition, Investigation, Resources, Supervision, Writing – review & editing. MS: Conceptualization, Investigation, Methodology, Software, Supervision, Validation, Visualization, Writing – original draft, Writing – review & editing, Funding acquisition.
