# Supplementary material for: Detecting T-cell clonal expansions and quantifying clone survival using deep profiling of immune repertoires
Source: Front Immunol. 2024 Apr 3;15:1321603. doi: 10.3389/fimmu.2024.1321603 (PMC11021634; doi:10.3389/fimmu.2024.1321603)
Supplement: Supplementary file 1 [file DataSheet_1.docx]

Supplementary Material

# Supplementary Figures

##
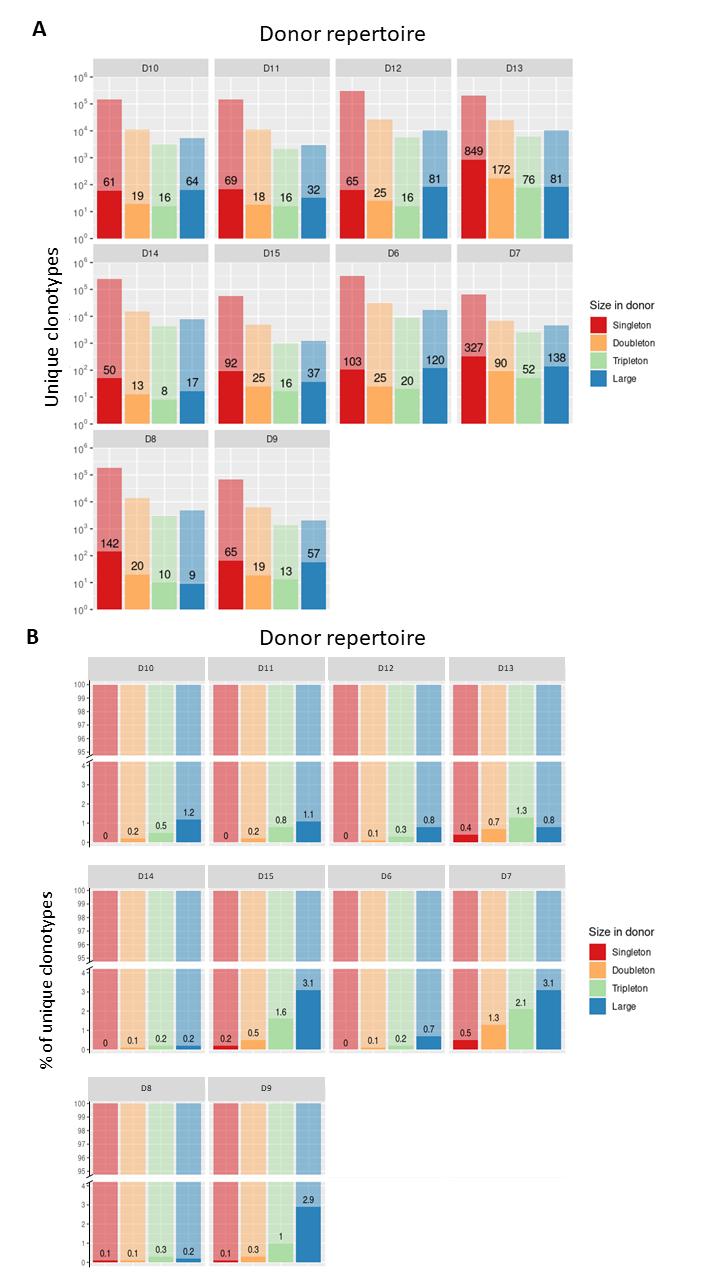
Supplementary Figures

**Supplementary Figure S1. Structure of donor repertoires (HSCT dataset).** Number (A) or percentage (B) of clonotypes grouped by clonotype size. Brighter color indicates the number/percentage of donor clonotypes that were also detected in the corresponding recipient repertoire. Note the y-axis break between 4%-95% on panel B.

**
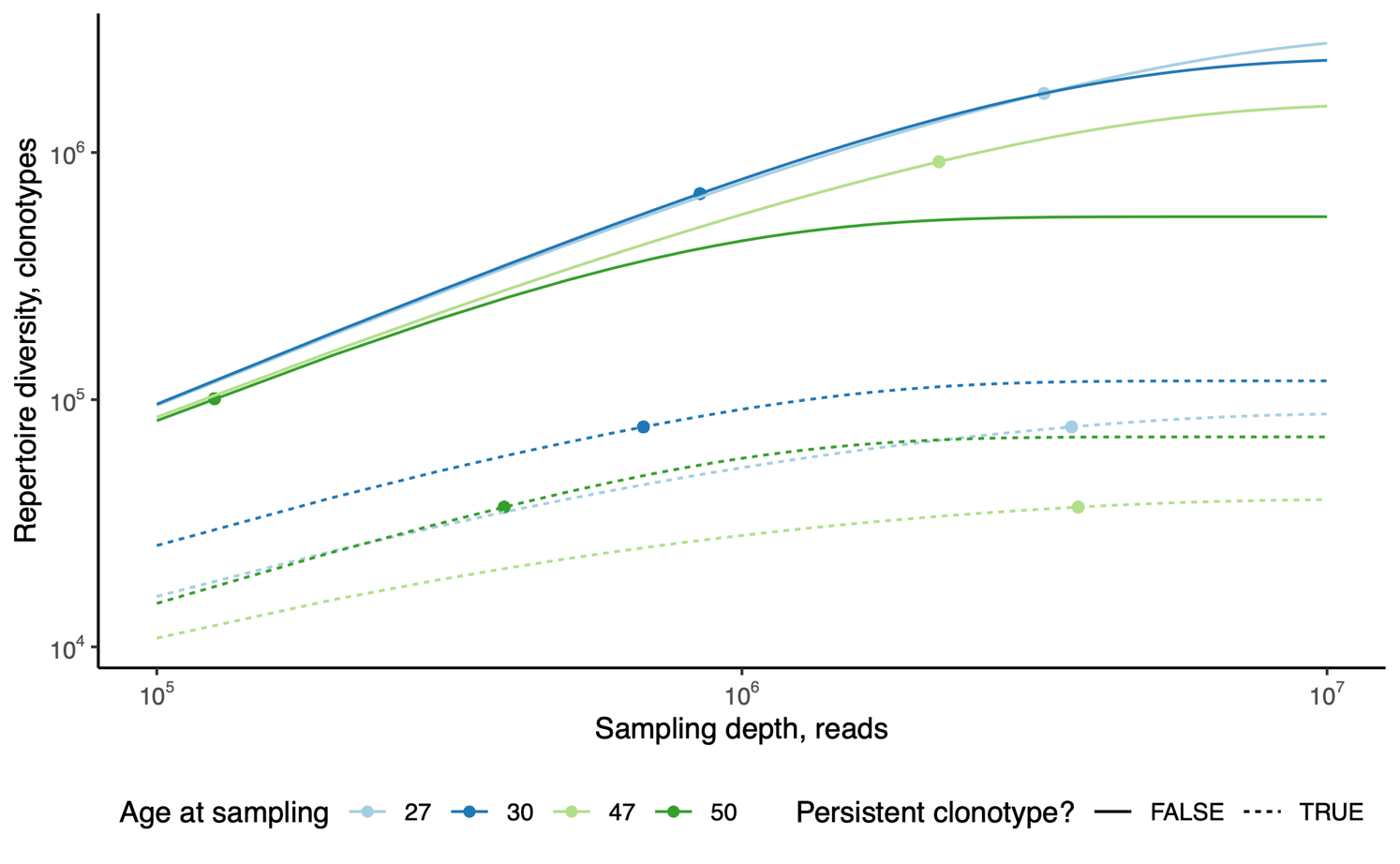
**

**Supplementary Figure S2.** Rarefaction analysis of donor repertoires sampled at 27 and 30 years (donor 1) and 47 and 50 years (donor 2). Number of clonotypes captured at a given sequencing depth is shown with interpolation and extrapolation performed according to the Poisson model (Coleman rarefaction). Persistent clonotypes that are found in both time points are shown with dashed lines, clonotypes captured at only one time point are shown with solid lines. Dots show observed repertoire diversity and sample size.


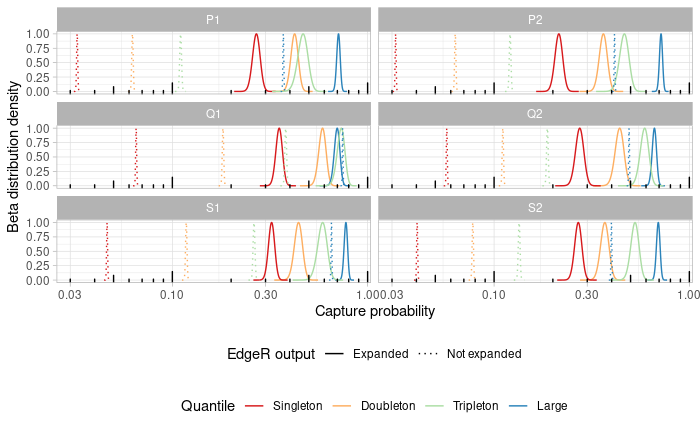


**Supplementary Figure S3.** Clonotype recapture probability for the clonotypes identified as significantly expanded and non-expanded by edgeR approach. Beta approximation for the distribution of capture probability at day 45 (“post” time point) of TCRs identified at day 15  (“pre” time point). TCRs annotated as significantly expanded (log fold-change > 5, p-value < 0.01, solid line (6)) are compared to the rest of TCR clonotypes in day 15 repertoire (dashed line). Clonotypes were grouped based on their size in “pre” time point: singletons, doubletons, tripletons and large clonotypes are supported by 1, 2, 3, and 4+ UMIs, correspondingly.
